# Cumulative radiation exposure from imaging procedures and associated lifetime cancer risk for patients with lymphoma

**DOI:** 10.1038/srep35181

**Published:** 2016-10-17

**Authors:** Grete Fabritius, Gunnar Brix, Elke Nekolla, Stefan Klein, Henning D. Popp, Mathias Meyer, Gerhard Glatting, Claudia Hagelstein, Wolf K. Hofmann, Stefan O. Schoenberg, Thomas Henzler

**Affiliations:** 1 Institute of Clinical Radiology and Nuclear Medicine, University Medical Center Mannheim, Medical Faculty Mannheim, Heidelberg University, Germany; 2 Federal Office for Radiation Protection, Department of Medical Radiation Protection, Neuherberg, Germany; 3 Department of Hematology and Oncology, University Medical Center Mannheim, Medical Faculty Mannheim, Heidelberg University, Germany; 4 Medical Radiation Physics/Radiation Protection, University Medical Center Mannheim, Medical Faculty Mannheim, Heidelberg University , Mannheim, Germany

## Abstract

The aim of this study was to systematically evaluate the cumulative radiation exposure and the associated lifetime-cancer-risk from diagnostic imaging in patients with Hodgkin-lymphoma-(HL) or diffuse-large-B-cell-lymphoma (DLBCL). 99 consecutive patients (53-males) diagnosed with HL or DLBCL were included in the study and followed. Based on the imaging reports, organ and effective-doses-(ED) were calculated individually for each patient and the excess lifetime risks were estimated. The average ED in the first year after diagnosis was significantly different for men (59 ± 33 mSv) and women (744 ± 33 mSv)-(p < 0.05). The mean cumulative ED in each of the following 5 years was 16 ± 16 mSv without significant differences between men and women-(p > 0.05). Over all years, more than 90% of the ED resulted from CT. The average cumulative radiation risk estimated for the first year was significantly lower for men (0.76 ± 0.41%) as compared to women (1.28 ± 0.54%)-(p < 0.05). The same was found for each of the subsequent 5-years (men-0.18 ± 0.17%; women-0.28 ± 0.25%)-(p < 0.05). In conclusion, for HL and DLBCL patients investigated in this study, a cumulative radiation risk of about 1 excess cancer per 100 patients is estimated for diagnostic imaging procedures performed during both the first year after diagnosis and a follow-up period of 5 years.

Imaging plays a pivotal role for staging, response evaluation, surveillance and prognosis in patients with malignant lymphoma^1^. Nowadays, computed tomography (CT) and hybrid imaging of ^18^F-fluorodeoxyglucose positron emission tomography and CT (^18^F-FDG-PET/CT) are the most widely used imaging procedures in these patients and are recommended by several guidelines^1^,^2^,^3^. However, CT and ^18^F-FDG-PET/CT lead to an exposure of patients to ionizing radiation associated with a cancer risk. Over the past decades, radiation exposure from medical imaging has significantly increased^4^,^5^. CT and nuclear imaging procedures like scintigraphy, single photon emission computed tomography (SPECT) and PET account for around half of the applied diagnostic radiation dose worldwide and for an even higher percentage in *first world* countries^5^. However, despite the still growing use of these radiation intense diagnostic procedures the awareness of health professionals regarding carcinogenesis associated with commonly performed imaging procedures is still deficient^6^. Although radiation exposures and risk from a single procedure is mostly negligible, the cumulative risk from multiple studies in particular in young and middle-aged cancer patients with a favorable prognosis is still a matter of concern^7^.

Classic Hodgkin lymphoma (HL) and non-Hodgkin lymphoma (NHL) account together for around 3% of all cancer cases (excluding non-melanoma skin cancer) in the more developed countries^8^. Due to considerable therapeutic advances - as for example intensive chemotherapy, stem cell transplantation, targeted therapies and intensity-modulated radiotherapy - cure rates and survival of patients with HL and NHL have significantly improved over the past two decades. These trends impose the requirement to minimize the detriment from diagnosis and treatment in order to prevent the risk for secondary malignancies.

The aim of this study was to evaluate the cumulative radiation exposure and the associated lifetime cancer risk resulting from staging, follow-up and surveillance of patients suffering from HL and diffuse large B-cell NHL (DLBCL).

## Materials and Methods

The Medical Ethics Commission II of the Medical Faculty of Mannheim approved the design of this HIPAA compliant study. Due to its retrospective nature, written informed consent was deemed not to be required.

### Patient selection and study design

The patient cohort investigated in this study included all patients with an age between 18–55 years diagnosed with HL or DLBCL in our university hospital center between 01/2008 and 12/2011 that underwent at least one X-ray or nuclear medicine examination. All patients were followed-up until 12/2013. All imaging procedures leading to a radiation exposure within two months before the date of diagnosis, as they presumably contributed to the diagnosis of cancer, and two to six years after diagnosis were taken into account. This led to an observation time of between 26 and 62 month, because all patients were observed until the end of 2013 regardless of the date of their diagnosis.

For each of the study participants, the following patient information and examination-specific data for every disease-related X-ray and nuclear medicine procedure were extracted from the hospital and radiology information systems:

- *Patient-specific data:* Identification code, date of birth, sex, date of assignment of the ICD-10 code (HL: C81.0–C81.9; DLBCL: C83.3), tumor histology, tumor stage at diagnosis, date of treatments and, if applicable, death.
- *Examination-specific data:* Date of examination, type of procedure and available dosimetric information:

￮ X-ray radiography and fluoroscopy: dose-area product 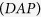,

￮ CT: volume CT dose index 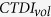 and dose-length product 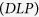,

￮ nuclear medicine: radiopharmaceutical and administered activity 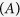


### Estimation of organ and effective doses

For each patient of the cohort (*i* = 1, …, *N*) and each type of procedure (*P*) organ doses 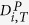 were estimated from the documented dose parameters 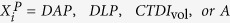 using tissue- (*T*) and sex-specific (*s*) dose coefficients 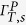.


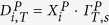


where the 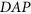 was not documented for a radiography, the corresponding national diagnostic reference level was used. Dose coefficients for radiographies were established with the program *PCXMC*^9^ using the settings for tube voltage and collimation given in national guidelines^10^. For CT scans, dose coefficients were determined using the program *CT*-*EXPO* (V2.0.1; Hamburg/Hannover, Germany^11^) from the respective *CTDI*_*vol*_ value and the scan length given by the ratio *DLP*/*CTDI*_*vol*_. For bone, thyroid and renal scintigraphy as well as for ^18^F-FDG-PET examinations, the dose coefficients provided in ICRP publication 80 were used^12^. Organ doses for combined ^18^F-FDG-PET/CT examinations were calculated separately for the CT and the PET part of the examination.

For each examination, the effective dose *E* was calculated from the organ doses using a radiation-weighting factor of *w*_*R*_ = 1 and the tissue-weighting factors *w*_*T*_ given in ICRP publication 103^13^. Based on the estimated doses per examination, the sum 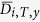 over the examination-specific organ doses 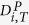 as well as the corresponding effective dose 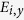 was computed for each patient and each year *y* = 1, …, 6 after diagnosis.

### Estimation of lifetime attributable risks

For a person *i* of sex *s*, exposed at age *e* to an annual cumulative organ dose 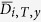, organ-specific lifetime attributable risk to develop cancer (cancer incidence) in the remaining life 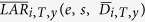, was estimated using the risk models developed by the BEIR VII committee^14^ assuming a linear non-threshold (LNT) dose-response relationship. Following the precautionary principle in medical radiation protection, and complying with the recommendation of the German Commission on Radiological Protection^15^, a dose and dose-rate effectiveness factor 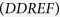 of 1 was applied. Risk estimates were adapted to the German general population. Details of the risk estimation are described in a previous publication^16^. The total risk 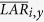 due to imaging procedures in year *y* was computed for each patient by adding up the organ specific risks 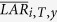.

### Data processing and statistical analysis

Analysis of the anonymized patient data was performed in the EXCEL environment (Microsoft Office EXCEL 2010; Microsoft Corporation, Redmond, WA), using the embedded programming language VBA. Statistical tests were done with the program JMP (JMP11; SAS Institute Inc., Cary, NC) at a significance level of *p* = 0.05.

To determine to what extent the annual organ doses, effective doses and radiation risks differ over the years *y* = 2–6 after diagnosis, a Kruskal-Wallis ANOVA test on ranks was performed. Since no differences were found over this period of time, non-zero dose and risk estimates were averaged over the years two to six and denoted by 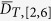, 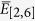, and 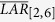.

To investigate age differences in the radiation exposure and cancer risk of patients, male and female patients were stratified into two age groups, namely ages 18–35 and 36–55 years. The significance of differences in the exposure and risk estimates between each of two specified patient groups (men vs. women, patiens with HL vs. patients with DLBCL, patients with lower tumor stage vs. patients higher tumor stage, younger vs. older patients) was evaluated by means of the Wilcoxon-Mann-Whitney-test.

## Results

**Figure 1:**
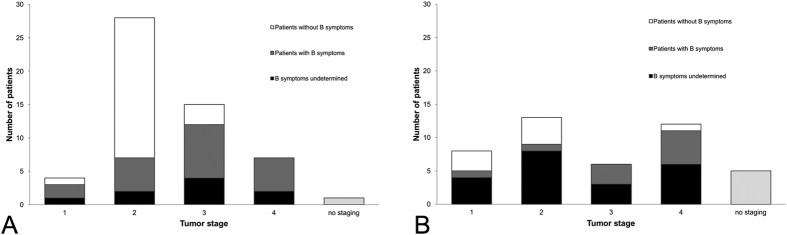
(**A,B**) Tumor-stage-specific composition of the patient cohort (according to the Ann-Arbor classification) for (**A**) Hodgkin lymphoma and (**B**) diffuse large B-cell lymphoma patients.

**Figure 2:**
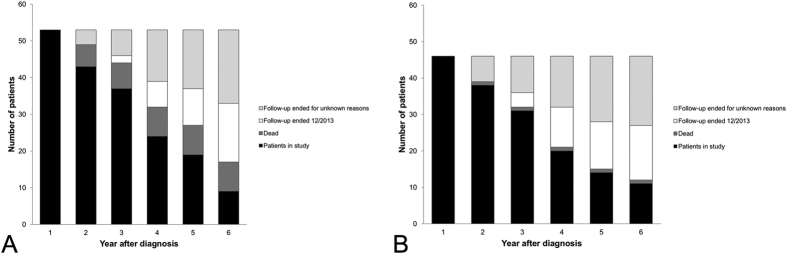
(**A,B**) Decrease of the number of patients over the follow-up period for (**A**) male and (**B**) female patients.

The study cohort comprises 99 patients (53 males, 46 females). 55 patients were diagnosed with HL and 44 with DLBCL. Differences in age at diagnosis between males and females were not significant (36.9 ± 10.0 years, range 18 to 55 years vs. 34.0 ± 11.6 years, range 18 to 55 years ). Patients with HL were significantly younger at diagnosis compared to patients with DLBCL (30.1 ± 8.9 years, range 18 to 48 years vs. 42.3 ± 9.1 years, range 18 to 55 years). Figure 1 summarizes the tumor stage according to the *Ann-Arbor* classification at diagnosis. With the exception of one patient who died before starting treatment, all patients were treated with either chemotherapy alone or combined radio/chemotherapy. Details are summarized in Table 1. In total, eight patients died, two of them suffered from HL, the others from DLBCL. 11 patients experienced a relapse of their disease. As already mentioned, patients in this study were not observed for the same follow-up period. The decrease in the number of patients over the years two to six for unknown reasons, death or end of the observation period (12/2013) is summarized in Fig. 2.

### Number, type and distribution of imaging procedures

In the 99 patients of the study cohort, a total of 2399 imaging procedures using ionizing radiation were performed. This corresponds to an average of 24.2 (SD: 13.1, range 1 to 78) examinations per patient, not taking into account the varying follow-up periods. 71.1% of all examinations were CT scans, 23.7% radiographies and 5.2% nuclear medicine procedures. In the first year after diagnosis a mean of 16.0 (SD: 8.1, range 1 to 55) procedures per patient were carried out whereas in each of the following years on average only 3.0 (SD: 3.8, range 0 to 24) examinations were performed per patient. Table 2 provides detailed data on the examinations performed in the first and the subsequent years.

In the first year after diagnosis, the number of all CT and radiographic examinations was slightly higher for women than for men, although not significantly (CT: 11.5 ± 4.4, range 5 to 21 vs. 10.4 ± 5.1, range 0 to 26; radiographies: 4.5 ± 5.6, range 0 to 33 vs. 3.9 ± 3.6, range 0 to 14). A significant difference was found only in the number of thoracic CT examinations, which was higher for women (5.0 ± 2.3, range 2 to 14 vs. 3.6 ± 1.8, range 0 to 10).

Furthermore, the number of CTs and radiographies was significantly lower for patients diagnosed with a lower tumor stage (Ann-Arbor stages 1 and 2) than for patients with a higher tumor stage (Ann-Arbor stages 3 and 4) (CT: 12.6 ± 4.8, range 4 to 23 vs. 10.2 ± 4.4, range 0 to 26; radiographies: 5.6 ± 5.9, range 0 to 33 vs. 2.9 ± 2.9, range 0 to 13) and lower for younger (18–35 years) than for older patients (36–55 years) (CT: 9.9 ± 3.5, range 0 to 17 vs. 11.8 ± 5.5, range 1 to 26; radiographies: 3.1 ± 3.4, range 0 to 14 vs. 5.2 ± 5.3, range 0 to 33). The number of nuclear medicine procedures was significantly higher for patients with Hodgkin lymphoma than for patients with B-Cell Lymphoma (1.0 ± 0.9, range 0 to 4 vs. 0.6 ± 0.7, range 0 to 2).

In the years 2 to 6 after diagnosis no differences in the number of any procedures were found for patients of different sex, age, diagnosis and tumor stage.

The patient with the highest number of examinations was a 38-year-old woman with HL. In this patient, a total of 78 imaging studies (44 CT scans, 33 radiographic examinations, one bone scintigraphy) were performed within a follow-up period of six years, with the majority of examinations (55) performed in the first year. Analysis of the patient record revealed that the high number of examinations was necessary due to poor therapeutic response, an iatrogenic pneumothorax and various other major clinical complications.

National and international guidelines for HL and DLBCL suggest a certain number of imaging procedures in the course of the disease as summarized in Table 3, but do not take into account additional interim examinations performed due to lack of remission or clinical complications. Accordingly, considerable more imaging studies were performed in our patient cohort in particular in the first year after diagnosis than recommended by the German guidelines for initial workup and therapy monitoring (cf. Tables 2 and 3). Compared to the German guidelines, more studies than recommended were performed in the observed patient cohort in the first year. Since this guidelines recommend no diagnostic imaging using ionizing-radiation during the follow-up period at all, all investigations carried-out during this period (Table 2) are considered as additionally.

### Organ dose

**Figure 3:**
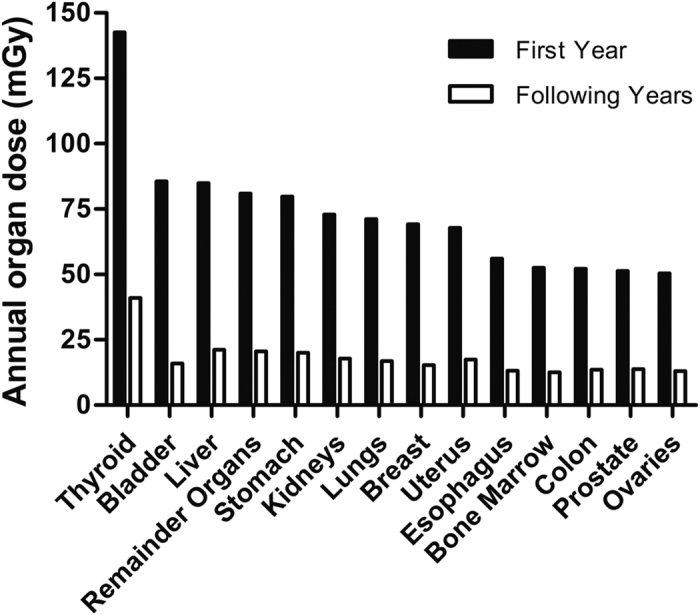
Estimated average organ doses cumulated over one year estimated for the first year after diagnosis and the following years. (Remainder tissues: adrenals, extrathoracic region, gall bladder, heart, lymphatic nodes, muscle, oral mucosa, pancreas, small intestine, spleen, thymus).

For both the first and the subsequent years, cumulative organ doses 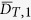 and 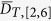 did not differ significantly between patients stratified by sex, age, tumor stage and diagnosis (*p* > 0.05). The cumulative organ dose values 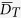 for the first year and the average annual doses for each of the subsequent years are shown in Fig. 3. The most highly irradiated organs in the first year were the thyroid gland (143 ± 89 mSv, range 0 to 506 mSv), the bladder (86 ± 59 mSv, range 0 to 262 mSv) and the liver (85 ± 46 mSv, range 0 to 251 mSv). In the subsequent years 2–6 the average annual cumulative dose 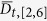 was highest to the thyroid gland (41 ± 36 mSv, range 0 to 139 mSv), the liver (21 ± 22 mSv, range 0 to 93 mSv) and the remainder tissues (21 ± 18 mSv, range 0 to 71 mSv). Remainder tissues are: adrenals, extrathoracic region, gall bladder, heart, lymphatic nodes, muscle, oral mucosa, pancreas, small intestine, spleen and thymus. For these organs, doses are averaged, as they have a relatively low susceptibility to ionizing radiation.

### Effective dose

The average cumulative effective dose 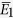 in the first year was significantly different for men (59 ± 33 mSv, range 0 to 153 mSv) and women (74 ± 33 mSv, range 17 to 186 mSv), whereas there were neither significant differences between patients with HL and DLBCL nor between patients in the two considered age groups or between different tumor stages. 92.7% of 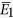 was caused by CT scans, 6.3% by nuclear medicine examinations and only 1, 0% by radiographies. In the subsequent years 2–6 no significant differences in the average annual effective dose between male and female patients, between diagnoses or tumor stages or between the two considered age groups were observed.

**Figure 4:**
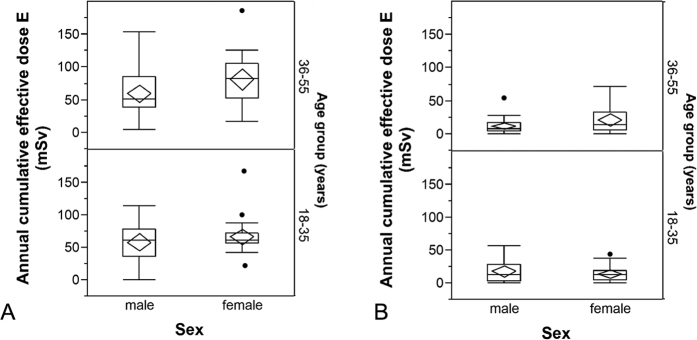
(**A,B**) Estimated average effective doses cumulated over one year in (**A**) the first year after diagnosis and (**B**) each of the following years stratified by sex and age. The horizontal line within the box represents the median value, the ends of the box the 75^th^ and 25^th^ percentiles. The whiskers extend from the ends of the box to the outer-most data point within the following distances: Upper Fence = upper quartile +1.5 × interquartile range, Lower Fence =lower quartile −1.5 × interquartile range, The dots represent outliers i.e. values outside this range. The confidence diamond within the box gives the mean and the upper and lower 95% confidence limits of the mean value.

The mean cumulative effective dose per year 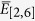 was 16 ± 16 mSv, range 0 to 71 mSv. Here, the relative contribution of CT scans was 97.5%. Nuclear medicine procedures and radiographies accounted for 1.7% and 0.8%, respectively. Figures 4A,B show the distribution of the annual effective dose in the first and the subsequent years stratified by sex and age group.

The highest effective dose in the first year (186 mSv) was estimated for the above-mentioned 38-year-old female patient undergoing the highest number of examinations. She also received the highest overall cumulative effective dose (426 mSv) for the entire period of observation, in her case six years.

### Lifetime attributable cancer risks

**Figure 5:**
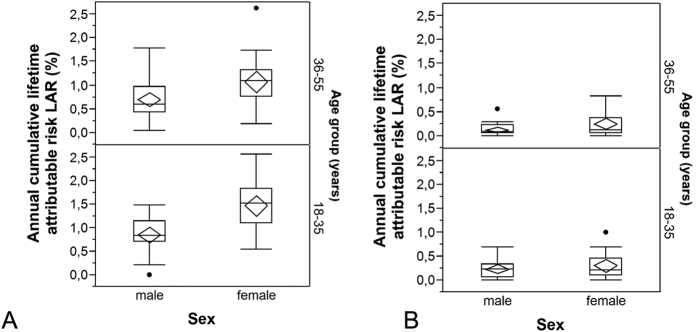
(**A,B**) Sex- and age-specific distribution of the average lifetime attributable risk of cancer incidence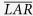 cumulated over on year in (**A**) the first year after diagnosis and (**B**) each of the following years. For details of presentation see Fig. 4.

The radiation risks estimated for imaging procedures performed during the first year after diagnosis 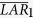 were significantly different for men and women (0.76 ± 0.41%, range 0.0 to 1.77% vs. 1.28 ± 0.54%, range 0.18 to 2.63%) as well as for younger and older patients (18–35 years, 1.18 ± 0.55%, range 0.0 to 2,56% vs. 36–55 years, 0.85 ± 0.49%, range 0.05 to 2.63%). Estimated 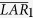 values are summarized in Fig. 5A,B, stratified by sex and age. Between patients with DLBCL and HL there were significant differences (0.85 ± 0.56%, range 0.0 to 2.56% vs. 1.13 ± 0.49%, range 0.2 to 2.62%), primarily due to differences in the age at diagnosis between the two groups. There were no differences between tumor stages. The radiation risks associated with imaging procedures performed on average per year in the subsequent years 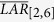 are also summarized in Fig. 5A,B. Significant differences were found between men (0.18 ± 0.17%, range 0 to 0.70%) and women (0.28 ± 0.25%, range 0 to 1.01%).

In men, colon, bladder and lungs accounted each for more than 10% to the annual radiation risks 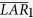 and 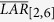. In women, breast, bladder and lungs each contributed more than 10% to the mentioned risk estimates. For both male and female patients, in the first as well as in the following years, the largest proportion of the annual radiation risk originates from radiation exposure of the remainder tissues (24.5% and 26.2%, respectively).

The patient with the highest overall 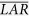 (summed over six years after diagnosis) was once again the 38-year-old female patient with HL who already had the most procedures and the highest cumulative effective dose. Her lifetime attributable risk of cancer incidence due to all procedures performed in the six years was estimated at 5.8%.

## Discussion

The presented retrospective patient study provides a detailed analysis of the individual cumulative radiation exposure and associated cancer risk resulting from diagnostic imaging procedures using ionizing radiation carried-out in patients with HL or DLBCL over a long oberservation period of up to 6 years. In contrast to a previous study that solely investigated the cumulative effective dose of CT and ^18^F-FDG-PET/CT examinations in patients with lymphoma over an average surveillance period of 8 months, the long observation period of our studies makes it possible to consider the entire follow-up period currently recommended by several guidelines (cf. Table 3)^17^.

The second advantage of our study design is that we included all diagnostic examinations using ionizing radiation over the whole observation period. Hereby, our results clearly demonstrate that patients undergo considerably more examinations when compared to recent guidelines (cf. Tables 2 and 3). Every procedure that was done exceeding these suggested numbers was considered as additional and due to complications. This was 40.3% of procedures in the first year and 100% of procedures in the following years, as german guidelines don’t recommend imaging using ionizing radiation in the follow-up period. This has to be accounted for when comparing the results with other studies. In contrast to guidelines that do not consider clinical complications, like atypical pneumonia that frequently occur during therapy and often lead to several thoracic CT examinations, our results provide a realistic scenario without any bias concerning the number, type and radiation dose of examinations clinically performed in patients with HL and DLBCL. Therefore, the cumulative effective doses estimated for the patients of our study cohort were markedly higher compared to those in a recently published study that used a Monte Carlo simulation to investigate radiation exposure and risk of adult patients with NHL associated with the imaging protocol of the HOVON 84 international multicenter trial^7^.

We deliberately considered only patients with HL or DLBCL with an age between 18–55 years to address the higher radiation risk of young and middle-aged patients. Moreover, elderly patients with both HL or DLBCL have also a considerably reduced disease-related overall survival rate when compared to young and middle-aged patients and thus a considerably decreased likelihood to develop a clinically manifest secondary cancer^18^. Within this context, it is important to consider that the minimum latency period to develop a secondary cancer, i.e. the period of time between radiation exposure and clinical manifestation of a secondary cancer, is assumed to be two to five years for leukemia and five to ten years for solid tumors.

In the patient cohort investigated in the present study, CT contributed to 93% of the cumulative effective dose within the first year after diagnosis and on average for 98% in each of the following years. Based on the *Lugano Classification*, ^18^F-FDG-PET/CT is nowadays considered as the first-line imaging modality for the initial staging as well as follow-up of patients with FDG-avid lymphomas whereas CT is recommended for all non FDG-avid lymphomas. A more widely use of hybrid ^18^F-FDG-PET/CT instead of CT alone as in our study will lead to an even higher radiation exposure in patients with FDG-avid lymphomas since the *Lugano Classification* recommends that hybrid imaging shall include a fully diagnostic contrast-enhanced CT.

Women in our patient cohort showed a significantly higher cumulative 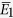 when compared to men in the first year after diagnosis. Since CT was the main source of ionizing radiation in this study, the higher 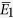 in women is most likely explained by a limited adaption of the individual CT scan protocol to the individual body size. Within this context, it is important to consider that over the last years several novel techniques for radiation dose reduction in CT - that were mainly not clinically available during our observation period - have been clinically implemented. Those techniques include more efficient X-ray detectors, iterative reconstruction techniques as well as automated tube current modulation and tube voltage selection based on the individual anatomy of the patient^19^,^20^. As one example out of many, a recently published study by Meyer *et al*. demonstrated that iterative reconstruction techniques allow for a 50% radiation dose reduction in whole-body staging examinations of patients with lymphoma^20^.

The interpretation of the cumulative effective dose estimated in this study has to consider that the effective dose characterizes the generic radiation risk of patients because neither the sex nor the age of the patients is considered and is thus not suitable for risk assessment of individual patients. Therefore, the ICRP stated that the effective dose should neither be used for epidemiological evaluations nor for detailed retrospective investigations of individual exposure and risks^13^. The effective dose was determined to be comparable to previous studies. The individual lifetime attributable risk 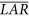 estimates computed in this study by using most recent organ-, sex- and age-dependent risk models yield a significantly higher cancer risk for women as compared to men. The higher risk in women can be explained by the higher radiation exposure estimated for women as compared to men as well as their higher risk coefficients for many organs and tissues, especially for breasts and lungs. The average 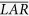 for men and women associated to the diagnostic imaging procedures considered in the present study corresponds to about 1 excess cancer in 100 lymphoma patients from diagnostic imaging performed in the first year after diagnosis (mean 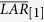 ≈ 1%), and to an additional excess cancer case for imaging procedures carried-out during a follow-up period of 5 years (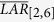 ≈ 0.23% per year). Compared to the lifetime baseline cancer risk (incidence excluding non-melanoma skin cancer) of a 35-year old man or woman in Germany of about 50 and 40%, respectively^21^, the average imaging related additional cancer risk estimated for the HL and DLBCL patients in the present study is relatively small, but not negligible mainly due to the low age of HL (mean, 30 years) and DLBCL patients (42 years) in our study cohort. It has to be noted that the reported risk estimates overestimate the real risks to some extent since they were derived using life table data for the entire german population and not data specific for lymphoma patients with a reduced life expectancy. For individual patients, the radiation risk from diagnostic imaging procedures can be considerably high. The highest risk of nearly 6% was estimated in case of the 38-year old female patient with HL undergoing a high number of CT scans due to clinical complications.

The estimated radiation risk of about 1% associated with diagnostic imaging procedures carried-out during a follow-up period of 5 years should be considered within the recent debate on the effectiveness of current imaging strategies to detect relapse in patients with lymphoma. One study that compared ^18^F-FDG-PET/CT against a combination of ultrasonography (US) and chest radiography for systematic follow-up of patients with high-risk HL found 97.5% of relapses using only US and chest radiography^22^. The estimated radiation dose in this study for a routine ^18^F-FDG-PET/CT follow-up examination was 14.5 mSv vs. 0.1 mSv for a chest radiography that was combined with US for follow-up. Thus, the authors concluded that US and chest radiography enable effective, safe, low-cost and especially low-risk routine surveillance imaging for patients at high risk of HL relapse^22^. Another recent study even suggested that routine, scheduled imaging might not be needed for follow-up of DLBCL, because the majority of relapses is detected outside of the planned follow-up examinations^23^. In this study, patient outcome did not differ between patients in which relapse was detected in routine follow-up examinations and patients with relapse outside of routine follow-up examinations^23^.

The present study has some potential limitations that need to be considered. First, our results are only representative for our university hospital in which mainly CT was used for staging and follow-up although ^18^F-FDG-PET/CT is nowadays recommended as the imaging gold-standard in case of patients with FDG-avid lymphomas. However, as already mentioned, radiation dose from hybrid ^18^F-FDG-PET/CT imaging comprising a fully diagnostic contrast-enhanced CT scan will lead to an even higher radiation dose when compared to CT alone. Second, this study includes solely data from the clinical and radiology information system of our university hospital center. Although most patients with HL and DLBCL receive their follow-up examinations at our institution, some patients may have received additional examinations outside of it. This may lead to a slight underestimation of the dose and risk estimates. Third, the results of this study are only representative for patients with HL and DLBCL with an age between 18–55 years. These patients were selected since their cure rate are generally good so that the risk to establish a clinically manifest secondary malignancy plays a pivotal role. The estimated radiation risks may thus not be directly transferable to elderly patients with aggressive types of lymphoma although imaging algorithms may not be different between patients with different types of lymphoma. Fourth, estimates of stochastic radiation risks are derived based on the LNT response model. Since experimental and radio-epidemiological studies do not provide conclusive evidence for the carcinogenicity of low levels of radiation (<about 50 mGy), there is a considerable controversy on the validity of the LNT model in the low-dose range^24^,^25^. Even for doses between 50 and 200 mSv, determined for the majority of the HL and DLBCL patients of our study cohort, the scientific evidence for carcinogenic radiation effects is still somewhat fuzzy^24^. Nevertheless, estimation of stochastic radiation risks associated with ionizing radiation by means of the LNT model is the most prudent and precautionary approach for radiation protection of patients^26^. All radiation risk estimates are, unquestionably, associated with uncertainties, as discussed in detail in the BEIR VII report^14^. In view of that, the report gives “subjective confidence intervals”. Referring to this concept, a 95% uncertainty range of a factor of 2 can be assumed for the *LAR* estimates given in this paper (95% *CI* = [0.5 * *LAR*; 2 * *LAR*]).

In conclusion, for the HL and DLBCL patients considered in the present study, a cumulative radiation risk of about 1 excess cancer per 100 patients is estimated for diagnostic imaging procedures performed during both the first year after diagnosis and a follow-up period of 5 years. Since CT is mainly responsible for the observed radiation exposure, novel CT techniques that enable significant dose reduction should be strictly implemented for imaging of patients with HL and DLBCL. Moreover, based on the results of novel studies that found most lymphoma relapses outside from routine follow-up examinations as well as a high accuracy and safety of US and chest radiography for the follow-up of patients with lymphoma, the overall usefulness of routinely performed follow-up CT or ^18^F-FDG-PET/CT examinations should be reevaluated in future guidelines.

## Additional Information

**How to cite this article**: Fabritius, G. *et al*. Cumulative radiation exposure from imaging procedures and associated lifetime cancer risk for patients with lymphoma. *Sci. Rep.* **6**, 35181; doi: 10.1038/srep35181 (2016).

## Figures and Tables

**Table 1:** Characteristics of the study cohort: number of patients, mean age at diagnosis, type of therapy, deaths and cancer recurrence.

	Number of Patients			Mean age at diagnosis (years)	Therapy (%)		Deaths (%)	Cancer recurrence (%)
	Total	Male	Female		Radio-chemotherapy	Chemotherapy only		
HL	55	29	26	30.1	66.7	33.3	3.6	9.1
DLBCL	44	24	20	42.3	40.9	59.1	10.9	13.6

Note: HL: Hodgkin lymphoma; DLBCL: Diffuse large B-cell lymphoma.

**Table 2:** Type and average number of procedures performed per patient within the first year after diagnosis and in each of the subsequent years.

Type of procedure^a^	Average number of procedures per patient and year	
	First year	Follow-up years
**CT**		
Head	0.34	0.01
Neck	2.59^c^	0.59
Thorax	4.24^c^	0.87
Abdomen	3.58^c^	0.70
Whole Body	0.20	0.04
**Total**	**10.95 (26)**^**b**^	**2.23 (11)**^**b**^
**Radiography**		
Skull, PA	0.08	0.01
Skull, LAT	0.01	<0.01
Chest, two planes	2.44	0.31
Chest, lying	1.41	0.22
Chest, one plane	0.01	0.02
Abdomen, AP	0.19	0.05
Abdomen, LAT	0.07	0.01
**Total**	**4.22 (33)**^**b**^	**0.60 (16)**^**b**^
**Nuclear Medicine**		
Tc-99m HDP (bone scintigraphy)	0.54	0.08
Tc-99m MAG3 (kidney scintigraphy)	0.03	<0.01
F-18 FDG-PET	0.27	0.04
**Total**	**0.84 (4)**^**b**^	**0.17 (3)**^**b**^
**Total amount**	**16.00**	**2.97**

Note: ^a^PA: Taken in direction posterior-anterior, AP: taken in direction anterior-posterior, LAT: taken from an lateral point of view, HDP: Hydroxydiphosphonate, MAG: Mercaptoacetyltriglycine, FDG: Fluorodeoxyglucose.

^b^Maximum number of procedures on one patient in parentheses.

^c^Overlapping CT scans of neck, thorax and abdomen acquired with different protocols in one session were counted as individual procedures and not as whole-body CT.

**Table 3:** Number and type of examinations recommended in German^2^,^3^, US^27^,^28^ and European guidelines;^29^,^30^.

**Country**	**Type of lymphoma**	**Initial workup**	**Number of examinations during**		
			**Therapy**	**Follow-up (5 years)**	**Refractory disease**
**Germany**	**HL**	1 Chest X-ray	2 CTs Neck/Thorax/Abdomen	Only in clinical relapse	1 CT Neck/Thorax/Abdomen
		1 CT Neck/Thorax/Abdomen			
	**DLBCL**	1 CT Neck/Thorax/Abdomen	1 CT Neck/Thorax/Abdomen, 1 PET/CT or CT Neck/Thorax/Abdomen^a^	Not in routine follow-up	None
**USA**	**HL**	1 Chest X-ray 1 PET/CT or CT Neck/Thorax/Abdomen	1-2 PET/CTs or CTs Neck/Thorax/Abdomen^b^	2-4 Chest-X-rays^b^ or CTs	1 PET/CT or CT
	**DLBCL**	1 CT Thorax/Abdomen and/or 1 PET/CT	2 PET/CTs or 1 PET/CT and 1 CT Neck/Thorax/Abdomen	0-4 CTs Neck/Thorax/Abdomen^c^	None
**Europe**	**HL**	1 Chest X-ray 1 PET/CT or CT Neck/Thorax/Abdomen	1 CT Neck/Thorax/Abdomen, 1 PET/CT or CT Neck/Thorax/Abdomen	Only if clinical symptoms occur	Not specified
	**DLBCL**	1 CT Neck/Thorax/Abdomen and 1 PET/CT	1 PET/CT, 1 CT Neck/Thorax/Abdomen or PET/CT	Not in routine follow-up	1 CT Neck/Thorax/Abdomen and 1 PET/CT

Note: ^a^PET/CT not paid by german statutory health insurance, otherwise PET/CT would be recommended; ^b^depending on tumor response; ^c^depending on initial stage.
