# Erratum: Cumulative radiation exposure from imaging procedures and associated lifetime cancer risk for patients with lymphoma

**DOI:** 10.1038/srep46644

**Published:** 2017-05-03

**Authors:** Grete Fabritius, Gunnar Brix, Elke Nekolla, Stefan Klein, Henning D. Popp, Mathias Meyer, Gerhard Glatting, Claudia Hagelstein, Wolf K. Hofmann, Stefan O. Schoenberg, Thomas Henzler

Scientific Reports 6: Article number: 35181; 10.1038/srep35181 published online: 10 17 2016; updated: 05 03 2017.

The original version of this Article contained a typographical error in the Abstract.

“The average ED in the first year after diagnosis was significantly different for men (59 ± 33 mSv) and women (744 ± 33 mSv)-(p < 0.05)”.

now reads:

“The average ED in the first year after diagnosis was significantly different for men (59 ± 33 mSv) and women (74 ± 33 mSv)-(p < 0.05)”.

This error has now been corrected in the PDF and HTML versions of the Article.

